# Powerful and interpretable behavioural features for quantitative phenotyping of *Caenorhabditis elegans*

**DOI:** 10.1098/rstb.2017.0375

**Published:** 2018-09-10

**Authors:** Avelino Javer, Lidia Ripoll-Sánchez, André E.X. Brown

**Affiliations:** 1 MRC London Institute of Medical Sciences, London, UK; 2 Institute of Clinical Sciences, Imperial College London, London, UK

**Keywords:** computational ethology, *C. elegans*, phenotyping, worm tracking

## Abstract

Behaviour is a sensitive and integrative readout of nervous system function and therefore an attractive measure for assessing the effects of mutation or drug treatment on animals. Video data provide a rich but high-dimensional representation of behaviour, and so the first step of analysis is often some form of tracking and feature extraction to reduce dimensionality while maintaining relevant information. Modern machine-learning methods are powerful but notoriously difficult to interpret, while handcrafted features are interpretable but do not always perform as well. Here, we report a new set of handcrafted features to compactly quantify *Caenorhabditis elegans* behaviour. The features are designed to be interpretable but to capture as much of the phenotypic differences between worms as possible. We show that the full feature set is more powerful than a previously defined feature set in classifying mutant strains. We then use a combination of automated and manual feature selection to define a core set of interpretable features that still provides sufficient power to detect behavioural differences between mutant strains and the wild-type. Finally, we apply the new features to detect time-resolved behavioural differences in a series of optogenetic experiments targeting different neural subsets.

This article is part of a discussion meeting issue ‘Connectome to behaviour: modelling *C. elegans* at cellular resolution’.

## 1. Introduction

Measuring phenotypes is essential in most areas of biology, but there are no rules that determine which aspects of a phenotype to focus on. This has led to calls for more exhaustive characterizations of phenotype under the umbrella term phenomics [1,2]. Imaging is well suited to phenomics because images can capture complex morphological differences and videos provide a natural extension to measure dynamics. However, the raw pixel intensities in images do not map directly to most quantities of interest and so an element of choice in representation remains. The success of deep learning approaches demonstrates the usefulness of automatically learned features on image analysis problems [3]. However, the tasks solved in deep learning have well-defined objectives such as minimizing cross-entropy loss. When the objective is scientific understanding or hypothesis generation, high performance depends not just on accuracy but on interpretability and the nonlinear combination of many features through neural networks does not typically lead to highly interpretable features. Our objective is to find a middle ground using a range of interpretable features optimized to quantify *Caenorhabditis elegans* morphology and behaviour.

In *C. elegans*, phenotyping morphology and behaviour, both of which are readily captured using imaging, have a long history dating back to Brenner's original paper describing the isolation of the first visible mutants [4]. Subsequent work by Croll and co-workers [5–9] pioneered the quantitative analysis of nematode behaviour in *C. elegans* and other species. A desire to increase throughput, sensitivity and the ability to quantify multiple phenotypes from the same recording have led to the development of many worm trackers over the intervening decades. Dusenbery [10] made a tracker in 1983 that could track the centroid position of 25 worms in real time at 1 Hz. The tracker was used to study oxygen and carbon dioxide responses and responses to a variety of chemicals [11].

The next generation of trackers were used to quantify speed [12] or the behavioural components of chemotaxis [13], still at relatively low resolution. High-resolution single-worm trackers were developed first simply to record single animals for long periods for subsequent manual annotation of egg laying [14], but were quickly adapted for use in high-dimensional quantitative phenotyping [15–19]. Throughput was increased by using multiple single-worm trackers in parallel [20]. At the same time, multi-worm trackers that tracked many animals at lower resolution were developed to increase throughput using a single camera [21,22]. Improvements in camera technology eventually led to the development of a high-resolution multi-worm tracker that operates in real time and records worm outline and skeleton at 30 Hz [23]. New trackers continue to be developed for specific applications or with new features [24–33]. We have also recently developed a high-resolution multi-worm tracker to store not just worm outline and skeleton but also worm pixels to achieve compression without losing information about worms and their surroundings [34]. Keeping a portion of the image data enables reanalysis using improved computer vision algorithms or manual annotation. In summary, there is no shortage of methods for collecting worm behaviour data and several options for quantifying behavioural phenotypes. Given the large set of possible approaches and features, a principled way of selecting useful features would be helpful.

In this paper, we introduce a set of handcrafted features that can be measured from single or multi-worm tracking data, provided there is sufficient resolution to quantify worm posture. The features are inspired by phenomics to be as exhaustive as possible, but with an explicit bias towards interpretability to support exploratory analyses, generate hypotheses and guide mechanistic studies. We use a large database of videos of mutant worms and wild isolates to select feature subsets that balance explanatory power and interpretability. We also analyse a new set of optogenetics experiments and show that the same feature set can be used to find differences in behavioural dynamics that reveal a range of behavioural responses to optogenetic stimulation of different neural circuits in worms.

## 2. Material and methods

The data from the mutants and wild isolates are from two previously published studies and are available online from the OpenWorm Movement Database community page on Zenodo https://zenodo.org/communities/open-worm-movement-database/. As described previously, the worms in these videos were young adults recorded for 15 min crawling on agar on a patch of *Escherichia coli* OP50 which serves as a food source [20,35]. Worms were allowed to habituate to the tracking plates for 30 min before recording.

The optogenetics experiments were performed on young adults on OP50 that were allowed to habituate for 30 min prior to recording. Worms were recorded for 7 min without perturbation and then stimulated with blue LEDs (peak intensity at 467 nm) with five 5 s pulses and one 90 s pulse, each separated by 60 s. Tracking plates were prepared with 300 µl OP50 liquid culture mixed with all-trans-retinal (ATR) dissolved in ethanol to a final plate concentration of 83 µM ATR (0.25 µl ATR each 300 µl of OP50). Control plates were prepared identically but using 100% ethanol without ATR. Plates were left for 48 h to dry with the lids on and stored for up to 5 days at 4°C. See table 2 for a list of strains used in the optogenetics experiments.

All worms were segmented, tracked and skeletonized using Tierpsy Tracker. [34] Binaries, source code and documentation are available at http://ver228.github.io/tierpsy-tracker/.

## 3. Results

### (a) Feature definition

For the initial parameterization of the worm, we focused on defining features that cover as much of the range of observable phenotypes as possible while remaining interpretable. We identified a range of feature classes that we then characterized by defining multiple features for each class to ensure phenotypic breadth. Following previous efforts at hand-crafting features for quantifying *C. elegans* [15–17,20,23,36], the classes cover morphology, path, posture, and relative and absolute velocities. Interpretability is subjective and depends on the assessor's background. As a rule of thumb, we considered more derived features—that is, features requiring more computational steps or longer algorithms to calculate—to be less interpretable. However, for the initial parametrization, we erred on the side of including more features because if a feature is subsequently found to be important for detecting strain differences, it could be worth sacrificing some interpretability.

**Figure 1.**
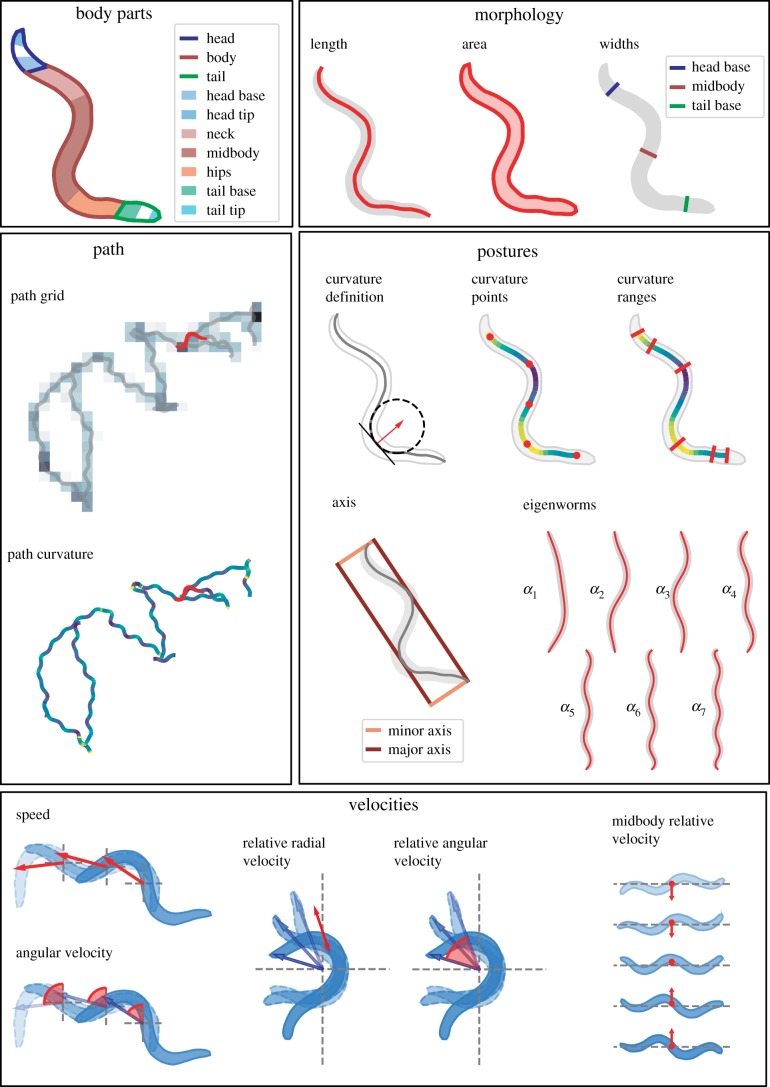
Schematics of the core features. Each of the features is summarized and expanded according to the transformations in figure 2. See the electronic supplementary material for detailed definitions.

The starting features are shown schematically in figure 1 and are described in more detail in the electronic supplementary material.

### (b) Feature expansion

**Figure 2.**
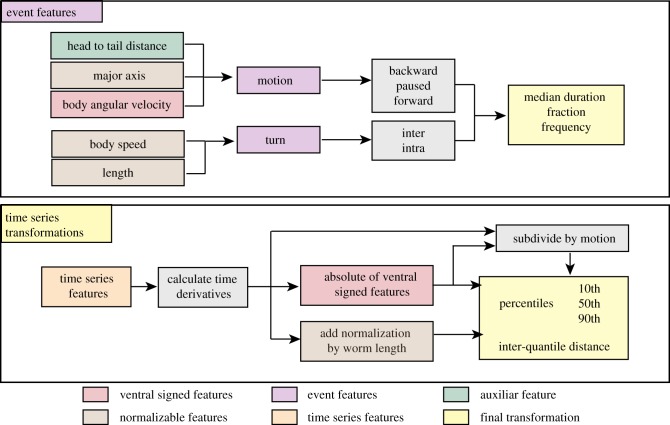
Operations that expand and summarize each of the core features. See electronic supplementary material, figure S1 for a more detailed description on how the core time series features are subdivided.

To further increase the breadth of phenotyping, we perform a series of operations that expands the total number of features (figure 2). First, any feature that can be localized to a part of the body, for example, curvature, is calculated separately for five segments along the worm (colloquially: head, neck, midbody, hips and tail). Velocities are calculated additionally at the head tip and tail tip because motion at the extremities, especially the head, is often informative. Second, we calculate the derivatives of any time series features (i.e. features that are calculated in each frame). For example, we calculate the rate of change of curvature for each segment. Third, we subdivide features according to motion state (forward, backward and pause) leading to features such as midbody curvature during reversals. Finally, the distributions of these features are quantified by calculating the 10th percentile, median and 90th percentile values.

The final phenotypic fingerprint thus derived has 4083 values. Although they are derived in several steps, they remain describable in words. For example, the feature *curvature_tail_w_forward_abs_90th* is the 90th percentile of the absolute value of the tail curvature measured while the worm is moving forwards. The large number of features belies an underlying simplicity: there are only 16 basic features, each subjected to similar operations during expansion.

### (c) Feature selection

Feature selection is useful to (i) remove noisy or irrelevant features and (ii) choose one from a set of highly correlated and therefore redundant features. Removing irrelevant features can improve performance while removing highly correlated features reduces the complexity of the representation without hurting performance. The features defined above involve relatively simple computations and were chosen based on our prior notions of what would be relevant for worm phenotyping so we do not expect many features to fall into the first category. On the other hand, the expansion procedure is likely to produce sets of correlated features that capture redundant information about the phenotype. In any case, relevance and redundancy are defined with respect to a particular dataset. We therefore chose to quantify the usefulness of the full set of features on a classification task on diverse previously published datasets [20,35] consisting of a total of 11 406 individual worms from 358 strains drawn from mutants affecting neurodevelopment, synaptic and extrasynaptic signalling, muscle function and morphology as well as wild isolates representing some of the natural diversity of *C. elegans* strains around the world.

As a prepossessing step, we cleaned the data by removing any feature assigned as not a number (NaN) in more than 2.5% of the worms in the full set. On this dataset, only paused motion state features were eliminated because in 14.3% of the videos, the worms never paused and therefore, the subdivision is not defined. Any remaining NaN values are imputed using the population mean value of the corresponding feature. We then *z*-normalize the data by subtracting the feature mean and dividing by its standard deviation to make features with different units comparable on the same scale.

**Figure 3.**
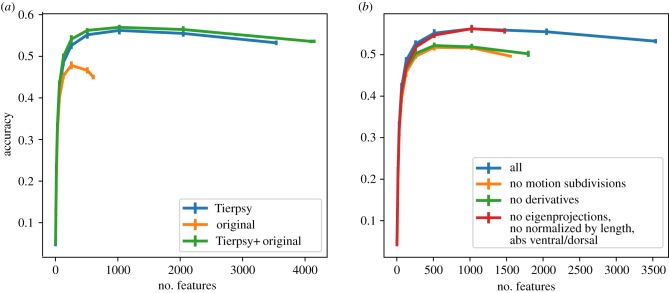
Results of recursive feature elimination. (*a*) The features reported in this paper (Tierpsy) achieve a higher accuracy than the ones used in Yemini *et al.* [20] (original). (*b*) The addition of transformations using subdivisions by motion type and derivatives over time are necessary to achieve the high accuracy. On the other hand, the eigenprojections, the normalization by length and the ventral/dorsal sign have little or no effect.

We divided the data into training and validation sets by randomly splitting the data per strain into 80–20% for training and testing, respectively. We then used recursive feature elimination to identify useful feature sets using the following procedure: (i) we fit a logistic regression model using stochastic gradient descent on the categorical cross-entropy loss. (ii) Each feature is ranked in importance by removing it from the fitted model and calculating the change in the loss. More important features will increase the loss when removed, while less important features will have little effect or even decrease the loss. (iii) The least important features are dropped until the next power of two is reached, e.g. if there are 3000 features, 952 features will be dropped leaving 2048, or 2^11^. We repeated this procedure 10 times for different random subsets of worms and plotted the classification accuracy as a function of feature number for our newly defined features (Tierpsy features), the previously defined features from Yemini *et al.* [20] and the combination of both feature sets (figure 3*a*).

The Tierpsy features perform better than the Yemini *et al.* features (peak accuracy of 56.21% at 1024 features compared to 47.82% at 256 features). The combined feature set shows the best performance, but the improvement over the Tierpsy features is small. This suggests that the Tierpsy features capture almost all of the phenotypic information in the Yemini *et al.* features as well as some new information that was missed. There is no drop in performance (and even a slight increase) as the first several thousand features are eliminated. The shape of the accuracy curve is highly reproducible when different subsets of worms are used for classification while the identity of the most useful features is highly variable, suggesting that many of the features in the total set are redundant. That is, within a set of correlated features, it makes little difference to classification accuracy which feature is kept and which are dropped.

The features that are selected also depend on nature of the strains used for feature selection. For example, if only wild isolate strains (rather than the full set of mutants which contain several strains with severe locomotion defects) are used for feature selection, the performance on classification on the mutant data is reduced (electronic supplementary material, figure S2). By contrast, if all strains or only mutants are used in feature selection, the performance on classifying wild isolates is unaffected. This may be because the nature of the variation between wild isolates is represented by the differences between some mutants, whereas there are differences between mutants that are no observed in wild isolates. This supports the choice of using a set of strains with a broad range of phenotypes in feature selection if the goal is to find a feature set that has the best chance of generalizing to unseen worm strains.

While classification accuracy does not allow us to prioritize features within correlated groups, interpretability can provide a guide. To bias the results towards simple interpretable features, we started by eliminating classes of features and steps in the feature expansion. We found that removing derivatives and the subdivision by motion state both significantly reduced classification accuracy confirming that these are useful operations (figure 3*b*). However, we found it was possible to eliminate eigenworm features and the normalization by worm length, and to use only the absolute value of features that had previously been signed as positive or negative based on dorsoventral orientation (e.g. curvature was originally defined as positive or negative depending on whether the body bend was dorsal or ventral). Together, removing these features reduces the total number by almost half with no detectable effect on accuracy. We label this reduced set of features the Tierpsy_2 k.

For accurate classification or for clustering applications where the full spectrum of differences and similarities would be useful, we recommend a reduced set of 256 features, which we label the Tierpsy_256 (electronic supplementary material, table S1), that balances completeness and compactness of the representation. Many phenotyping tasks occur on a smaller scale, with just a handful of strains compared to a reference (such as several mutants compared to a wild-type strain). If there are specific hypotheses for relevant phenotypic differences, having a large number of features to choose from makes it more likely that the hypotheses will be testable without having to code new features. However, for exploratory work, 256 features can lead to a larger number of differences than are needed to guide experiments and the large number of features increases the burden of multiple testing corrections. We have therefore used a combination of classification power, subjective interpretability and coverage of feature classes to define the Tierpsy_8 and Tierpsy_16 (table 1), which give classification accuracies of 20.37 ± 0.41% and 28.67 ± 0.45%, respectively (mean ± standard deviation).

**Table 1.** List of manually selected features. The first eight features correspond to Tierpsy_8, while the whole list corresponds to Tierpsy_16.

length_90th
width_midbody_norm_10th
curvature_hips_abs_90th
curvature_head_abs_90th
motion_mode_paused_fraction
motion_mode_paused_frequency
d_curvature_hips_abs_90th
d_curvature_head_abs_90th
width_head_base_norm_10th
motion_mode_backward_frequency
quirkiness_50th
minor_axis_50th
curvature_midbody_norm_abs_50th
relative_to_hips_radial_velocity_tail_tip_50th
relative_to_head_base_radial_velocity_head_tip_50th
relative_to_head_base_angular_velocity_head_tip_abs_90th

**Figure 4.**
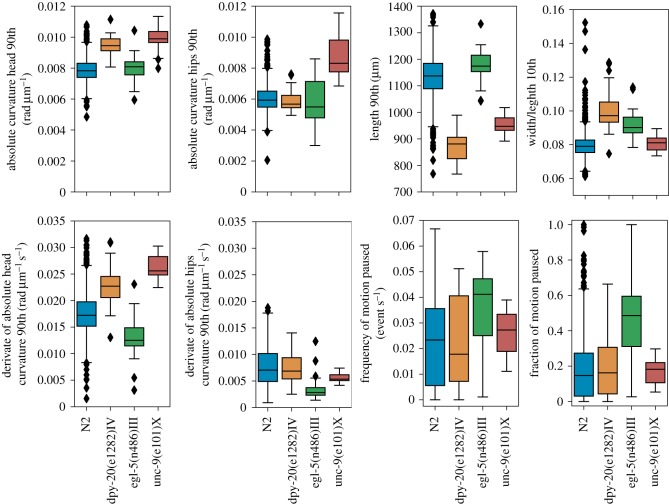
Boxplots of N2 and three mutants using the Tierpsy_8 subset of features. The small set of features facilitates a visual comparison between different strains.

Pre-selecting these smaller sets of features before performing a new analysis reduces the multiple testing burden and results in phenotypic fingerprints that can be visualized and understood at a glance (figure 4).

### (d) Direct analysis of time series for optogenetic experiments

The feature expansion and summarization above captures some dynamic aspects of phenotype, but is intended for comparisons where the relevant differences are not localized in time and could occur at any point during a recording. For optogenetic experiments where the stimulation has a clear start and stop, it makes more sense to align time series and look for differences directly rather than summarizing the entire experiment in a feature vector.

As above, we wanted to analyse data with a range of phenotypic differences. We therefore collected data from 11 strains expressing channel rhodopsin in different neural subsets (table 2). We separate the data for each video into short pulses (five 5 s long segments) and long pulses (one 90 s long segment) and calculate histograms for each feature in each set. The behaviour may vary during or after a pulse and therefore, it is useful to use multiple time bins to capture transient effects. We pooled the histograms for each strain with ATR and the controls without ATR. Because worms do not make ATR, any behavioural effects observed in the no-ATR condition are more likely to be generic blue light responses rather than ChR2-specific effects. The blue light response is most clearly observable during the 90 s pulses (electronic supplementary material, figure S3).

**Table 2.** List of strains used in optogenetic experiments, their genotypes and the neurons where channelrhodopsin is expressed.

strain	neuron	genotype (all found information)
AQ2028	CEP, ADE, PDE	lite-1(ce314); ljIs100 [dat-1::ChR2::YFP; Punc-122::GFP]
AQ2051	M3R, M3 L	lite-1 (ce314); Ex206 [eat-4::ChR2::YFP]
AQ2052	ASH, ASI, PVQ	lite-1 (ce314); ljIs105 [sra-6::ChR2::YFP, Punc-122::GFP]
AQ2232	AVM, ALM, PVM, PLM	lite-1 (ce314); ljIs111 [mec-4::ChR2]
AQ2235	ADF, ASH, AWC, PHA, PHB	lite-1 (ce314); ljIs114 [sra-6::FTF::ChR2::YFP, gpa-13::FLPase]
AQ3071	HSN, GLR	RQ10; wzIs6 [egl-6a::ChR2::YFP]
HBR180	AVA, AVD, AVE, AVB, PVC	goeIs25 [nmr-1::ChR2::mKate2-unc-54-3'utr, unc 119 (+)] [suspected]
HBR187	AVA, AVD, AVE, AVB, PVC	goeIs28 [nmr-1::ChR2::mKate2-unc-54-3'utr, unc 119 (+)] [suspected]
HBR222	AVM, ALM, PVM, PLM	goeIs43 [mec-4::ChR2::mKate2-unc-54-3'utr, unc 119 (+)]
HBR520	RIS	goeIs101 [aptf-1-5'utr::ChR2::mKate2-aptf-1-3'utr, unc119(+)]
MW544	AWB	raxIs15 [str-1::ChR2::sI2GFP]

**Figure 5.**
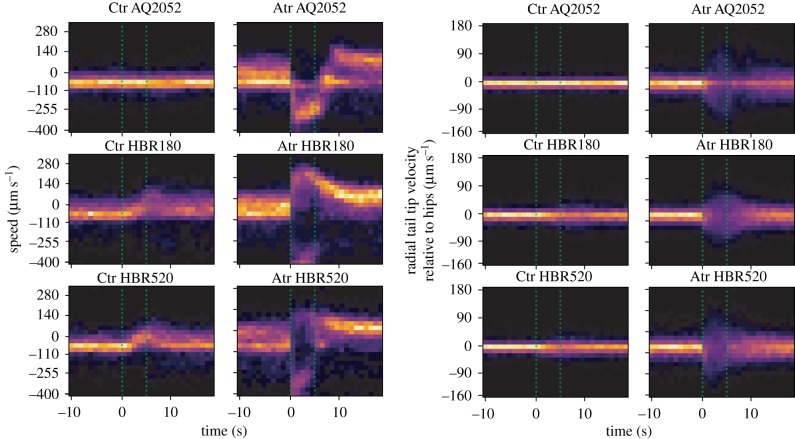
Example of two-dimensional histograms of short pulses for different strains and features.

**Figure 6.**
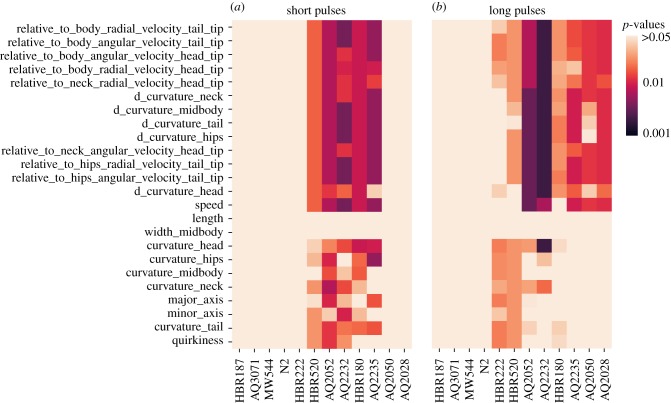
Comparisions between the changes of behaviour among the different strains under blue light stimulation for selected features. The heatmaps show the *p*-values of the Jensen–Shannon divergence between ATR and control plates for the short pulse experiments (*a*), and the long pulse experiments (*b*). The *p*-values were corrected for multiple comparisons within strains using the Benjamini–Hochberg procedure.

A selection of responses to 5 s optogenetic activation are shown in figure 5. There are clear differences between treatment and controls in several features for the three strains expressing ChR2 in different neural circuits. To systematically find features that respond differently to blue light between treatment and no-ATR controls, we calculated the Jensen–Shannon divergence between the treatment and control distributions for each feature and used a permutation test to determine a *p*-value for the comparison. Finally, we corrected for multiple comparisons within a given strain using the Benjamini–Hochberg procedure to control the false discovery rate at 0.05 [37]. The results are summarized in figure 6. Only five strains show *p*-values smaller than 0.05 after correction (AQ2235, AQ2052, AQ2232, HBR180, HBR520) in the short pulses (figure 6*a*).

On the other hand, three additional strains show differences (HBR222, HBR520, AQ2050, AQ2028) when analysing the long pulse data (figure 6*b*), suggesting a slower response to optogenetic stimulation in these strains. Finally, it is worth noting that the three strains that did not show a significant difference compared to the control do not have a *lite-1* deletion mutation, so it is possible that an underlying optogenetic effect is masked by their aversive blue light response.

**Figure 7.**
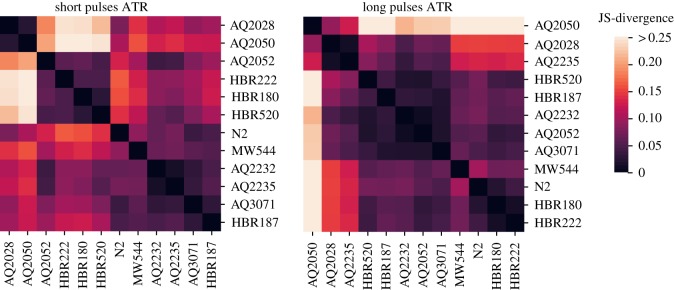
Cluster maps of the median value of the Jensen–Shannon divergence between different strains among the ATR plates.

The permutation tests show that behaviour is significantly affected by optogenetic stimulation in several strains, but do not show how the features change to allow a comparison between strains. In order to quantitatively compare the behavioural responses between strains, we calculated a distance matrix between each strain in each condition (control and ATR) using the Jensen–Shannon divergence among the corresponding feature histograms. The results for the samples with ATR are shown in figure 7. Two-thirds of the strains are close to N2 in the long pulses but fewer than half are close to N2 in the short pulses. The observed clustering pattern suggests that there is a range of distinct behavioural phenotypes induced by the optogenetic stimulation of different neural subsets. The equivalent plots for the control plates are shown in electronic supplementary material, figure S4.

## 4. Discussion

The features we have defined here are intended to provide a range of options to balance power and interpretability in behaviour representation from a small set of easily interpretable features to a large set of features that provide improved classification accuracy compared a previously defined set of handcrafted features. Using a diverse set of worm behaviour data from mutants and wild isolates, we found, as expected, that our feature definitions led to groups of correlated features that contained redundant information. We took advantage of this redundancy to favour interpretable features over equally useful but less interpretable features.

We found that two categories of features could be eliminated entirely with little effect on classification accuracy: those derived from the distribution of eigenworm amplitudes and those based on dorsoventral asymmetries. There is no contradiction between the elimination of eigenworm features and their usefulness in other applications [19,29,38,39]. The implication of this result is simply that the information present in the distribution of the eigenworm amplitudes taken across an entire video is captured using other more interpretable features. The eigenworm representation remains useful for many other applications, especially where an understanding of postural time series is important, rather than phenotypic summary based on posture distributions calculated for an entire video. Similarly, the known asymmetry between dorsal and ventral turns will clearly be important in some studies. It is just that on average, for distinguishing worm strains, it is not a critical distinction and most of the information is present in the symmetrized data. This is a positive result for multi-worm tracking data where the dorsoventral orientation of worms is difficult to determine. Our results suggest that the Tierpsy features will be as useful for high-resolution multi-worm tracking as they are for single-worm tracking.

The data we used to perform feature selection cover a range of mutant phenotypes and the natural variation of wild isolates. Our goal was that the feature subsets we selected will be useful for capturing as complete a range of phenotypic variation as possible (electronic supplementary material, figure S2). For applications where interpretability is paramount, they provide a useful starting point. However, for any new application with a different kind of phenotypic variation such as new mutants or worms in different experimental conditions, a different subset of features could be more appropriate. Therefore, for applications where there are sufficient data to use a training set on feature selection and a hold-out set for testing, we would recommend repeating the feature selection procedure starting from the full set of features or the Tierpsy_2 k. Alternatively, if prior knowledge or specific hypotheses suggest a certain class of features is important, manually selected features can be simply added to one of the smaller feature subsets to capture the relevant effect without unduly increasing the burden of multiple testing.

## 5. Conclusion

A critical step in any phenotyping project is choosing the right representation for the problem at hand. Phenomics is based on the assumption that the right representation can be difficult to determine *a priori*, and that it is therefore useful to measure phenotypes as exhaustively as possible [1,2]. We have adopted this approach to define a large number of behavioural features that are then selected based on how well they explain data from a diverse set of strains and based on a subjective assessment of their interpretability. The direct parameterization of behaviour we describe here is just one approach to the larger problem of the quantitative analysis of behaviour characteristic of computational ethology [40–42]. We have found that this approach has reasonably good power to detect subtle behavioural differences and is particularly useful in cases where interpretability is paramount.

In this paper, we have focused on applications of phenotyping to analyse mutant strains, wild isolates and optogenetically stimulated animals, but the same features could be useful in phenotypic drug screens, to quantify behavioural declines with ageing, or to characterize disease models.

One of the reasons the phenomic approach to behaviour analysis is useful is that animal behaviour is complex and the effects of perturbations are difficult to predict. The same is increasingly true for artificial agents controlled by artificial neural networks, which has led to calls for an ethology of artificial agents to understand their behaviour [43]. When applied to understanding the output of increasingly detailed simulations of *C. elegans* [44], we believe that a high-dimensional representation of behaviour will be essential to provide enough constraints to fit model parameters that are difficult to estimate directly from experiments. We propose that the features defined here are a useful starting point for performing quantitative model validation for cellular-level simulations based on the *C. elegans* connectome.

## Supplementary Material

Detailed feature description and supplementary discussion.

## Data accessibility

This article has no additional data.
