# Supplementary material for: Powerful and interpretable behavioural features for quantitative phenotyping of Caenorhabditis elegans
Source: Philos Trans R Soc Lond B Biol Sci. 2018 Sep 10;373(1758):20170375. doi: 10.1098/rstb.2017.0375 (PMC6158219; doi:10.1098/rstb.2017.0375)
Supplement: Detailed feature description and supplementary discussion. [file rstb20170375supp1.pdf]

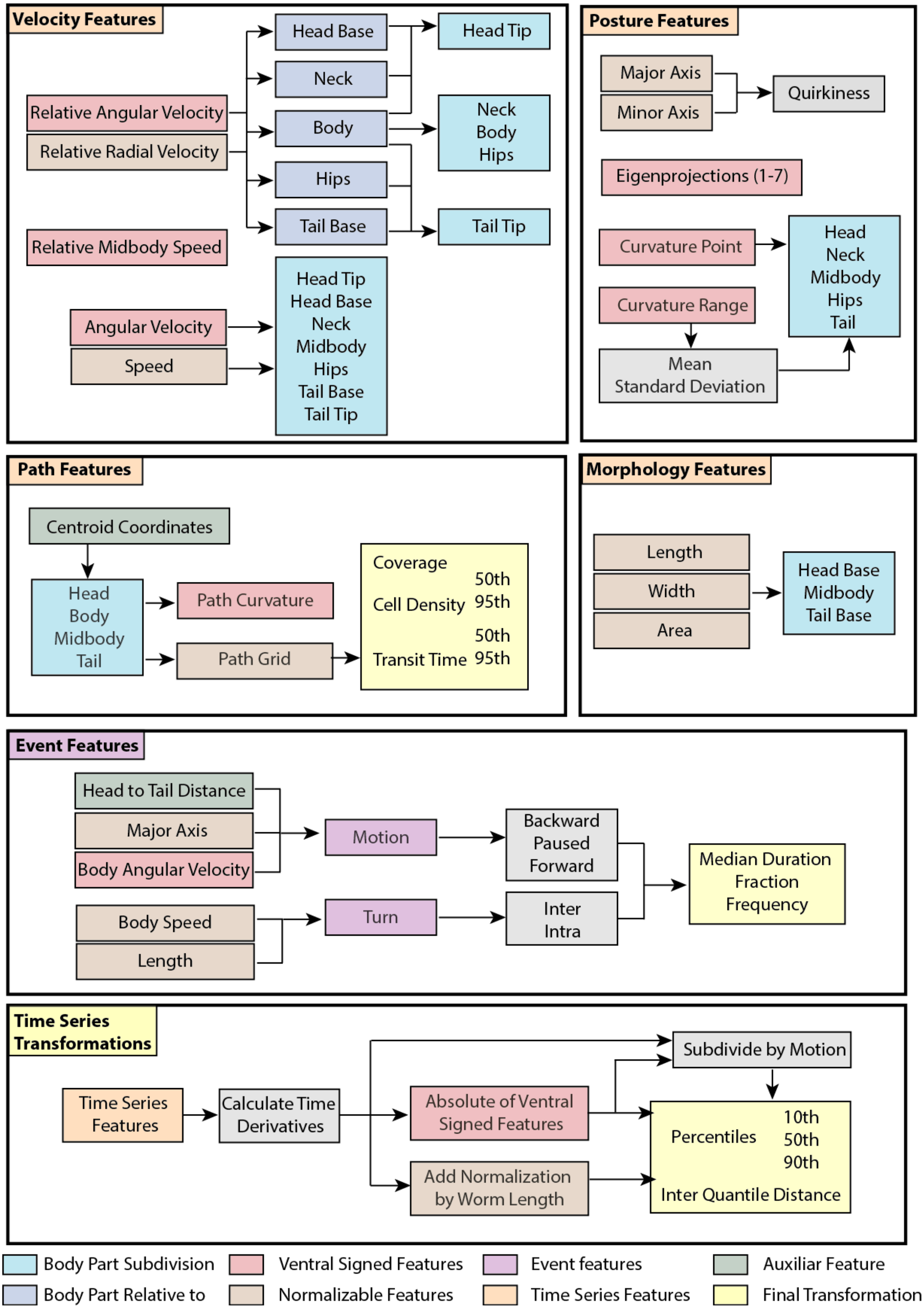

Figure S1: Operations that expand and summarize each of the core features.

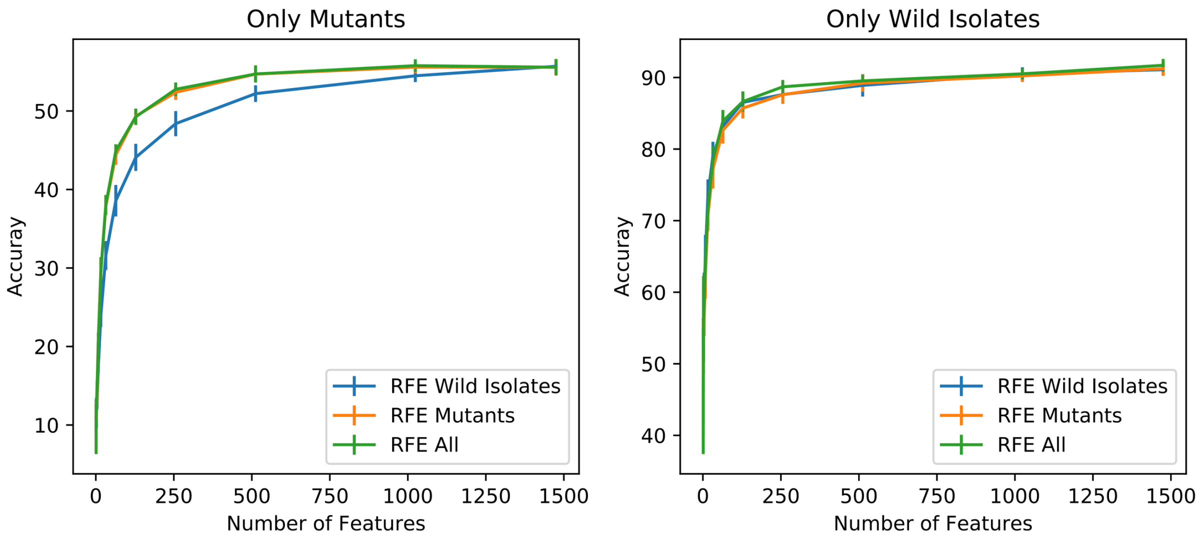

Fig. S2: Comparison of classification performance using different strain subsets for feature selection using recursive feature elimination (RFE). Each curve shows the results using features selected using all strains, mutant strains without wild isolates, or wild isolates only. The plot on the left shows the classification performance of each feature subset on the mutant data. The plot on the right shows the classification performance of each feature subset on the wild isolate data. The features selected using only wild isolate data perform worse on classifying mutants, while the features selected using mutants perform as well as features selected directly on wild isolates for classifying wild isolates.

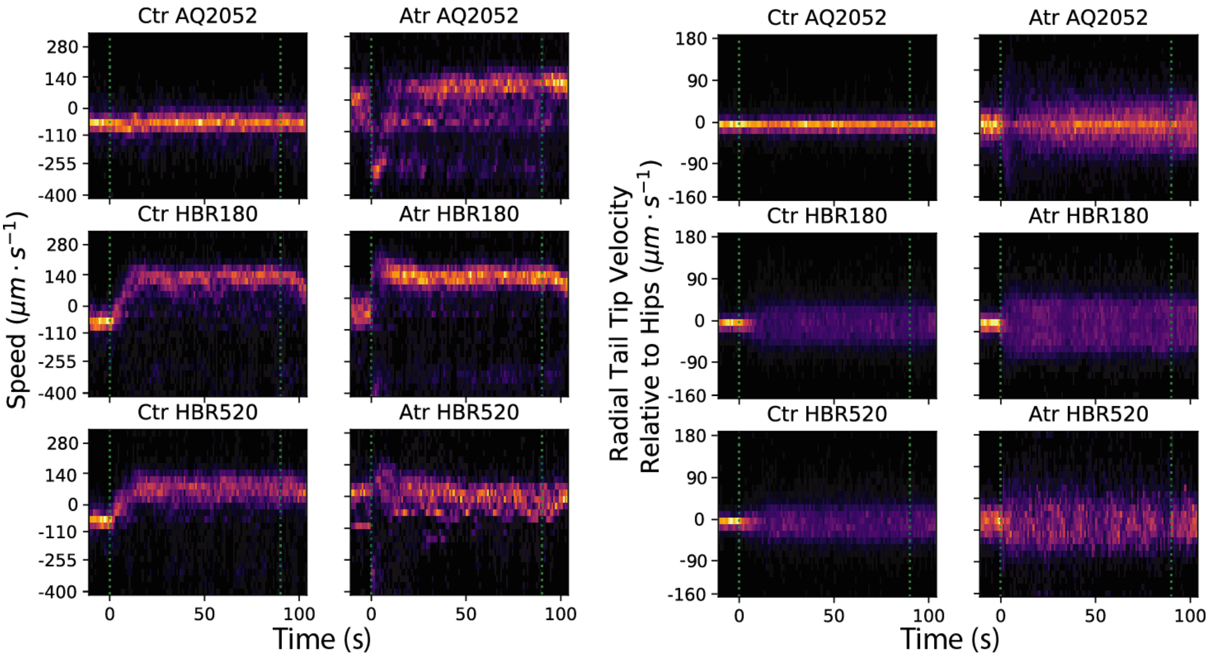

Figure S3: Example of 2D histograms of long pulses for different strains and features.

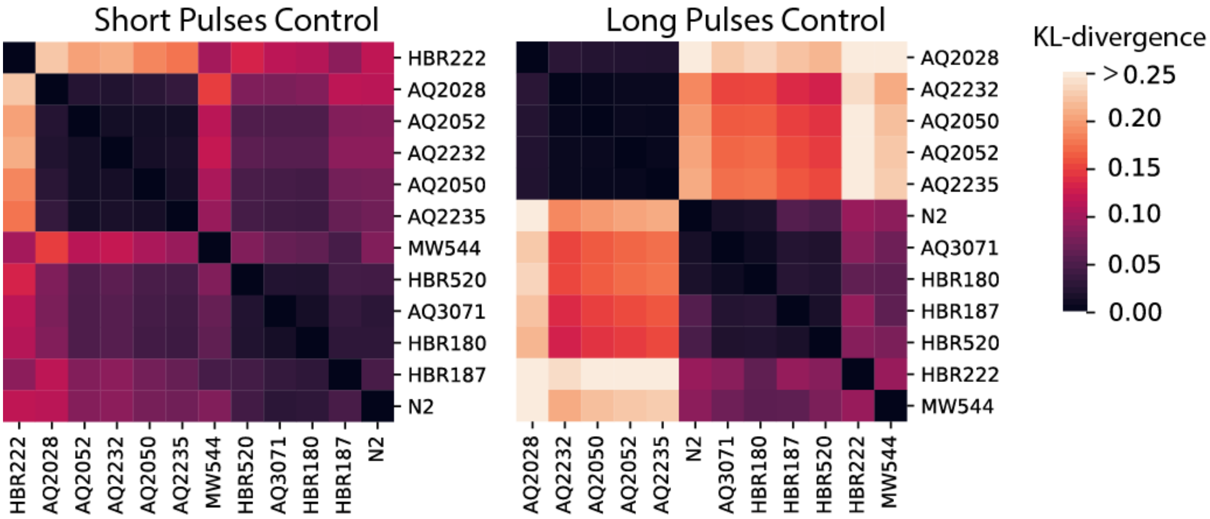

Figure S4: Cluster maps of the median value of Jensen-Shannon divergence between different strains for the control plates.

Features Definitions

Body Parts

The default skeleton is contains 49 points equally distributed along the length of the worm body. If the number of segments is different, the corresponding indices will be calculated as $[\frac{i}{49}N]$. The segment limits of each body part are shown in the table below.

Part	Lower Index	Upper Index
Head	1	8
Body	9	41
Tail	42	49
Head Tip	1	3
Head Base	4	8
Neck	9	16
Midbody	17	33
Hips	34	41
Tail Base	42	44
Tail Tip	45	49

Morphology

Length

The length of a skeleton with n points p is defined as the sum of all the euclidean distances between two consecutive points in the skeleton as:

$$L = \sum_{i=1}^{n-1} \parallel \overrightarrow{p_{i+1}} - \overrightarrow{p_i} \parallel$$

Area

The area is calculated from the worm contour using the shoelace method defined as:

$$A = \frac{1}{2} \left| \sum_{i=1}^{n-1} x_i \, y_{i+1} + x_n y_1 - \sum_{i=1}^{n-1} x_{i+1} \, y_i - x_1 y_n \right|$$

where x and y are the coordinates of a non-crossing contour with n elements. This method will give incorrect results for coiled (self-intersecting) worms shapes, but these are not currently skeletonised by the current algorithm.

Widths

Median contour width at specific parts of the body. The width at each point of the skeleton must be given as an input by the user (this is calculated in an earlier processing step by Tierpsy Tracker).

Postures

Curvature

The curvature κ is defined as the rate of change of a curve's tangent angle with respect to its arclength and is given by

$$\kappa = \frac{|x'y'' - y'x''|}{(x'^2 + y'^2)^{\frac{3}{2}}}$$

where x and y are the coordinates of each point p. We calculate the numerical derivatives as

$$x_i{}' = \frac{(x_{i+\delta} - x_{i-\delta})}{2\delta}.$$

In order to obtain a smoother gradient, we use a $\delta = 2$.

Finally, for each curvature along the skeleton we summarize the data using:
- The curvature at specific points in the curve.
- The mean and the standard deviation along specific body part ranges.

Major axis, minor axis and quirkiness

We use the OpenCV function *minAreaRect* to calculate the minimum-area bounding rectangle, and define the smallest and largest sides of the rectangle as the minor a and major A axis respectively.

We define quirkiness as

$$Q = \sqrt{1 - a^2/A^2},$$

making it a metric analogous of the eccentricity but using the bounding box axes instead of the ones of a fitted ellipse.

Eigenworms coefficients

Following the procedure of Stephens *et al.* [1], the frame of reference is changed by calculating the tangent angle between consecutive points as

$$\theta'_i = \arctan(x_{i+1} - x_i, y_{i+1} - y_i)$$
$$\theta_i = \theta'_i - \frac{1}{N_s - 1}\sum_{n=1}^{N_s-1} \theta'_i,$$

where N_s is the total number of segments in the skeletons, x_i and y_i are the segment coordinates, and θ_i is the corresponding segment angle. The final features are coefficients of the angles projected onto the eigenworms as

$$a_\mu = \sum_i u_\mu \theta_i$$

where μ is the eigenworm number and u are the eigenworms previously calculated using principal components analysis on the data in Yemini *et al.* [2]. We use the first 7 eigenworms, which capture more than 98% of the variance in the original dataset.

Velocities

All the velocities are calculated using a user defined time window Δt with a default value of $1/3s$.

Speed and Angular Velocity
For each body part segment, we calculate its centroid

$$\vec{c} = \frac{1}{m-l}\sum_{i=l}^{m} \vec{p_i}$$

and its orientation vector

$$\vec{o} = \vec{p_m} - \vec{p_l}$$

where l and m are respectively the lower and upper indices of a given body part, and $\vec{p}$ are the coordinates of the skeleton.

The angular velocity is calculated as

$$\theta = \arctan(o_x(t), o_y(t))$$

$$\omega(t) = \frac{\theta(t) - \theta(t+\Delta t)}{\Delta t}$$

For the speed, we first calculate the centroid c velocity as

$$\overrightarrow{v(t)} = \frac{\overrightarrow{c(t)} - \overrightarrow{c(t+\Delta t)}}{\Delta t}.$$

and then calculate the signed speed as

$$v(t) = |\overrightarrow{v(t)}|\text{sign}(\overrightarrow{v(t)} \cdot \overrightarrow{o(t)})$$

so the speed is positive when the worm is moving forwards and negative when it is moving backwards.

Relative Velocities

The centres of mass are calculated as above. We then shift the coordinate system of a given body part $\overrightarrow{c_b}$ to be centred with respect to a reference body part $\overrightarrow{c_r}$ as

$$\overrightarrow{c_b^r} = \overrightarrow{c_b} - \overrightarrow{c_r}.$$

The angular and radial velocity are calculated by changing from the cartesian (x, y) to the polar coordinate system (ϕ, r). The relative angular velocity is the derivative with respect to time of the ϕ coordinate of $\overrightarrow{c_b^r}$, while the relative radial velocity is the same but for coordinate r.

The relative midbody velocity is a special case where b is the midbody and r is the body, and the time derivative is calculated with respect to the cartesian coordinate x.

Path

The path features with respect to a given body part are calculated over the centre of mass of the corresponding skeleton segment as above.

Path Curvature

We resample the path over space so each point in the trajectory is equidistant, and then calculate the curvature equation described in the Curvature section. Finally, we interpolate curvature back so each element is equally separated in time.

Path Grid

The path grid is a 2D histogram where each cell represents a small region in the recording area. The counts represent the amount of time (in frames) spent in a given cell. Each side of the individual cells has a default value $250\mu m$. From this grid the following features are extracted:

Path Coverage is total area explored by the worm, calculated as the total number of grids explored by the worm times the individual grid area.

Path Density is the probability of a worm staying in a given cell calculated as the counts per cell over the total number of counts. Only the 50th and 90th percentiles of the density distributions are calculated.

Grid Transit Time is the time spent in a cell before moving to the next one. Only the 50th and 90th percentiles of the transit time distributions are calculated.

Transformations

Time derivative of a given time series feature are calculated over a window with a default value of 1/3*s*, similar to the velocity.

Percentiles: 10th, 50th and 90th percentiles of a given time series feature.

Interquantile Range (IQR): calculated as the 75th minus the 25th percentile of a given time series distribution. It is a robust statistic of the distribution spread.

Normalization by Worm Length: the units of a given feature are converted from μm to body lengths.

Absolute Ventral/Dorsal Sign: the value of a feature is signed according to the ventral/dorsal orientation [2].

Subdivision According to Movement: a time series is subdivided into segments where the worm is moving either forward, backwards, or is paused. These movement states are determined from the worm velocity.

References

1. Stephens GJ, Johnson-Kerner B, Bialek W, Ryu WS. 2008 Dimensionality and Dynamics in the Behavior of C. elegans. *PLoS Computational Biology* **4**, e1000028. (doi:10.1371/journal.pcbi.1000028)

2. Yemini E, Jucikas T, Grundy LJ, Brown AEX, Schafer WR. 2013 A database of Caenorhabditis elegans behavioral phenotypes. *Nature Methods* **10**, 877–879. (doi:10.1038/nmeth.2560)

Table S1: There are many sets of 256 features that perform similarly in classifying the strains used in this paper. For this larger set, we have not applied an interpretability criterion. Nonetheless, for concreteness and reproducibility, we include one instance, which we label the Tierpsy_256 here.

motion_mode_paused_frequency
motion_mode_paused_fraction
curvature_std_neck_abs_90th
d_curvature_neck_w_backward_abs_90th
angular_velocity_tail_tip_w_backward_abs_IQR
curvature_std_midbody_abs_10th
relative_to_head_base_radial_velocity_head_tip_50th
width_tail_base_w_forward_50th
minor_axis_w_forward_10th
d_relative_to_head_base_angular_velocity_head_tip_w_backward_abs_IQR
d_relative_to_body_radial_velocity_head_tip_w_forward_90th
relative_to_head_base_angular_velocity_head_tip_abs_50th
relative_to_body_radial_velocity_tail_tip_w_backward_10th
d_angular_velocity_tail_base_w_backward_abs_90th
relative_to_body_radial_velocity_hips_w_forward_90th
width_head_base_w_forward_10th
curvature_std_tail_w_forward_abs_90th
d_area_w_backward_10th
d_curvature_mean_hips_w_forward_abs_IQR
relative_to_head_base_radial_velocity_head_tip_w_forward_10th
width_midbody_10th
curvature_head_abs_90th
d_curvature_std_head_w_forward_abs_50th
curvature_mean_head_abs_90th
curvature_std_neck_w_forward_abs_50th
d_width_head_base_IQR
d_minor_axis_w_backward_90th
length_w_backward_10th
width_midbody_w_forward_10th
motion_mode_forward_fraction
curvature_std_head_w_forward_abs_50th
relative_to_neck_radial_velocity_head_tip_50th
motion_mode_backward_frequency
curvature_std_neck_abs_10th
angular_velocity_abs_IQR
length_IQR
d_angular_velocity_head_tip_abs_10th
relative_to_hips_radial_velocity_tail_tip_50th
d_speed_head_tip_50th
relative_to_head_base_radial_velocity_head_tip_w_forward_90th
quirkiness_50th
relative_to_head_base_radial_velocity_head_tip_w_backward_90th
d_width_tail_base_IQR
minor_axis_w_forward_90th
d_relative_to_neck_radial_velocity_head_tip_w_forward_90th
d_relative_to_tail_base_radial_velocity_tail_tip_50th
curvature_head_w_forward_abs_IQR
relative_to_head_base_radial_velocity_head_tip_w_backward_IQR
d_path_curvature_midbody_abs_90th
relative_to_body_radial_velocity_head_tip_w_forward_50th

d_relative_to_head_base_radial_velocity_head_tip_w_backward_50th
d_length_50th
width_midbody_90th
curvature_hips_w_backward_abs_50th
path_transit_time_body_95th
d_length_w_forward_IQR
motion_mode_backward_fraction
speed_head_tip_w_forward_IQR
curvature_std_hips_abs_10th
relative_to_head_base_angular_velocity_head_tip_abs_IQR
d_major_axis_w_backward_50th
d_curvature_std_tail_w_forward_abs_90th
curvature_mean_hips_w_backward_abs_90th
path_coverage_head
d_relative_to_tail_base_angular_velocity_tail_tip_w_forward_abs_90th
d_relative_to_head_base_angular_velocity_head_tip_w_backward_abs_90th
relative_to_head_base_radial_velocity_head_tip_w_forward_IQR
width_tail_base_w_backward_IQR
curvature_mean_neck_abs_50th
curvature_std_neck_w_forward_abs_10th
curvature_std_midbody_w_backward_abs_10th
d_major_axis_50th
curvature_hips_abs_90th
relative_to_body_radial_velocity_head_tip_50th
curvature_tail_w_backward_abs_90th
curvature_mean_head_w_forward_abs_50th
d_width_midbody_w_forward_10th
width_midbody_w_backward_50th
relative_to_head_base_radial_velocity_head_tip_10th
curvature_midbody_abs_90th
curvature_tail_abs_90th
curvature_mean_neck_w_forward_abs_IQR
width_tail_base_w_backward_10th
turn_intra_duration_50th
d_relative_to_head_base_angular_velocity_head_tip_w_forward_abs_90th
width_midbody_w_backward_10th
d_curvature_std_head_w_forward_abs_10th
curvature_mean_tail_abs_90th
speed_10th
d_angular_velocity_head_tip_w_forward_abs_90th
relative_to_body_radial_velocity_hips_50th
d_width_tail_base_50th
width_tail_base_10th
d_speed_head_tip_w_forward_50th
d_angular_velocity_head_tip_w_forward_abs_50th
d_width_head_base_w_backward_50th
speed_head_tip_10th
curvature_std_head_abs_IQR
curvature_hips_w_backward_abs_IQR
d_width_midbody_50th
d_speed_head_tip_w_backward_50th
length_w_forward_IQR
width_tail_base_w_backward_50th
curvature_tail_abs_10th
d_width_head_base_w_forward_IQR

d_curvature_std_midbody_w_backward_abs_10th
d_width_head_base_w_forward_50th
width_head_base_10th
d_length_w_backward_IQR
d_curvature_std_hips_w_forward_abs_90th
motion_mode_backward_duration_50th
d_curvature_std_head_abs_90th
d_length_w_backward_10th
curvature_std_midbody_w_backward_abs_50th
curvature_std_midbody_w_backward_abs_90th
relative_to_neck_radial_velocity_head_tip_w_forward_10th
curvature_std_hips_w_backward_abs_IQR
d_path_curvature_tail_w_backward_abs_90th
curvature_head_w_forward_abs_50th
relative_to_body_radial_velocity_neck_50th
relative_to_neck_radial_velocity_head_tip_w_forward_50th
d_relative_to_body_radial_velocity_tail_tip_w_backward_10th
width_head_base_w_forward_50th
curvature_mean_tail_w_backward_abs_90th
curvature_midbody_w_forward_abs_50th
d_relative_to_head_base_radial_velocity_head_tip_50th
length_w_backward_IQR
d_relative_to_neck_radial_velocity_head_tip_w_forward_50th
d_angular_velocity_midbody_w_backward_abs_90th
d_curvature_std_head_w_backward_abs_50th
curvature_std_hips_abs_50th
width_tail_base_90th
relative_to_body_radial_velocity_tail_tip_50th
angular_velocity_head_base_abs_10th
curvature_std_hips_w_backward_abs_90th
d_relative_to_head_base_radial_velocity_head_tip_w_backward_10th
path_curvature_body_abs_10th
speed_w_forward_90th
d_relative_to_body_radial_velocity_neck_w_forward_90th
d_relative_to_body_radial_velocity_tail_tip_w_backward_50th
d_angular_velocity_hips_w_backward_abs_90th
d_relative_to_body_angular_velocity_hips_w_forward_abs_10th
width_midbody_w_forward_50th
d_curvature_mean_tail_w_forward_abs_10th
d_quirkiness_50th
speed_hips_w_backward_90th
d_curvature_hips_w_backward_abs_90th
curvature_mean_tail_w_backward_abs_IQR
d_curvature_mean_hips_w_backward_abs_90th
angular_velocity_tail_base_w_backward_abs_90th
d_width_head_base_50th
path_transit_time_midbody_95th
path_curvature_midbody_w_backward_abs_10th
speed_w_forward_IQR
width_midbody_50th
d_curvature_neck_w_backward_abs_10th
d_curvature_mean_neck_w_backward_abs_90th
relative_to_hips_radial_velocity_tail_tip_w_forward_IQR
angular_velocity_head_tip_w_forward_abs_50th
d_curvature_mean_head_w_forward_abs_10th

curvature_std_neck_abs_50th
angular_velocity_head_base_abs_50th
curvature_std_midbody_w_backward_abs_IQR
d_curvature_std_neck_w_backward_abs_10th
speed_tail_base_w_forward_IQR
angular_velocity_tail_base_w_forward_abs_50th
d_relative_to_body_radial_velocity_tail_tip_w_forward_IQR
relative_to_tail_base_radial_velocity_tail_tip_50th
d_angular_velocity_midbody_w_backward_abs_50th
curvature_std_neck_w_backward_abs_50th
width_midbody_w_backward_90th
d_relative_to_body_radial_velocity_hips_50th
curvature_std_neck_w_backward_abs_10th
d_relative_to_body_radial_velocity_tail_tip_w_forward_90th
d_curvature_head_w_backward_abs_50th
angular_velocity_head_base_w_forward_abs_10th
d_relative_to_body_angular_velocity_neck_w_backward_abs_10th
d_relative_to_body_angular_velocity_tail_tip_abs_10th
d_curvature_mean_midbody_abs_90th
width_tail_base_w_backward_90th
curvature_std_midbody_abs_90th
d_curvature_midbody_w_backward_abs_10th
curvature_tail_w_forward_abs_90th
curvature_std_midbody_abs_50th
angular_velocity_head_base_w_forward_abs_50th
speed_head_tip_w_forward_50th
d_curvature_head_w_forward_abs_10th
angular_velocity_head_tip_w_backward_abs_IQR
d_curvature_hips_w_backward_abs_10th
d_relative_to_body_radial_velocity_neck_w_forward_10th
d_relative_to_tail_base_radial_velocity_tail_tip_w_forward_10th
minor_axis_10th
speed_head_tip_w_forward_10th
d_relative_to_body_angular_velocity_hips_w_backward_abs_90th
d_curvature_mean_hips_abs_IQR
relative_to_tail_base_radial_velocity_tail_tip_90th
relative_to_neck_radial_velocity_head_tip_w_forward_90th
d_curvature_mean_hips_abs_90th
curvature_hips_abs_10th
d_major_axis_w_forward_IQR
d_angular_velocity_head_base_w_forward_abs_90th
curvature_std_midbody_abs_IQR
relative_to_tail_base_radial_velocity_tail_tip_w_backward_90th
curvature_midbody_abs_IQR
speed_head_tip_w_backward_10th
curvature_std_head_abs_90th
width_midbody_w_forward_90th
d_angular_velocity_midbody_w_backward_abs_IQR
d_curvature_hips_abs_90th
d_length_w_backward_90th
d_curvature_mean_head_w_backward_abs_50th
relative_to_body_radial_velocity_head_tip_w_backward_50th
relative_to_neck_radial_velocity_head_tip_w_forward_IQR
d_curvature_mean_head_w_forward_abs_50th
d_angular_velocity_abs_10th

angular_velocity_head_base_w_backward_abs_90th
d_major_axis_w_forward_10th
curvature_tail_w_forward_abs_10th
d_length_10th
d_curvature_std_hips_w_backward_abs_IQR
d_angular_velocity_head_tip_abs_90th
d_length_90th
d_angular_velocity_tail_base_w_backward_abs_50th
d_curvature_std_head_abs_IQR
curvature_mean_hips_w_backward_abs_IQR
speed_tail_base_w_backward_90th
angular_velocity_head_base_w_backward_abs_IQR
relative_to_neck_radial_velocity_head_tip_w_backward_50th
d_minor_axis_50th
d_relative_to_tail_base_radial_velocity_tail_tip_w_backward_90th
angular_velocity_head_tip_abs_50th
motion_mode_forward_frequency
width_tail_base_50th
d_curvature_mean_neck_w_forward_abs_IQR
angular_velocity_tail_base_w_forward_abs_IQR
curvature_std_head_abs_50th
speed_hips_w_forward_IQR
d_angular_velocity_tail_base_w_backward_abs_IQR
d_relative_to_body_radial_velocity_head_tip_w_backward_10th
d_relative_to_body_radial_velocity_tail_tip_w_forward_10th
d_curvature_mean_neck_w_backward_abs_10th
d_relative_to_head_base_radial_velocity_head_tip_w_forward_IQR
relative_to_neck_angular_velocity_head_tip_w_backward_abs_10th
relative_to_head_base_radial_velocity_head_tip_w_backward_10th
d_length_w_backward_50th
curvature_hips_abs_IQR
d_curvature_std_neck_w_backward_abs_50th
d_angular_velocity_neck_w_forward_abs_10th
d_speed_neck_50th
d_relative_to_body_radial_velocity_neck_w_backward_50th
d_major_axis_w_backward_10th
d_curvature_std_head_w_backward_abs_10th
d_path_curvature_midbody_w_backward_abs_90th
d_relative_to_body_angular_velocity_neck_w_backward_abs_90th
d_relative_to_hips_radial_velocity_tail_tip_50th
width_tail_base_w_forward_10th
